# Challenges of Mechanochemistry: Is In Situ Real‐Time Quantitative Phase Analysis Always Reliable? A Case Study of Organic Salt Formation

**DOI:** 10.1002/advs.201700132

**Published:** 2017-05-08

**Authors:** Adam A. L. Michalchuk, Ivan A. Tumanov, Sumit Konar, Simon A. J. Kimber, Colin R. Pulham, Elena V. Boldyreva

**Affiliations:** ^1^ REC‐008 Novosibirsk State University ul. Pirogova 2 630090 Novosibirsk Russian Federation; ^2^ EaStChem School of Chemistry and Centre for Science at Extreme Conditions (CSEC) University of Edinburgh Edinburgh EH9 3FJ UK; ^3^ EPSRC Centre for Continuous Manufacturing and Crystallisation (CMAC) Joseph Black Building, King's Buildings, David Brewster Rd. Edinburgh EH9 3FJ UK; ^4^ Institute of Solid State Chemistry and Mechanochemistry SB RAS Kutateladze 18 630128 Novosibirsk Russian Federation; ^5^ European Synchrotron Radiation Facility 71 avenue des Martyrs 38000 Grenoble France

**Keywords:** kinetics, mechanochemistry, organic salts, X‐ray powder diffraction

## Abstract

Mechanochemical methods offer unprecedented academic and industrial opportunities for solvent‐free synthesis of novel materials. The need to study mechanochemical mechanisms is growing, and has led to the development of real‐time in situ X‐ray powder diffraction techniques (RI‐XRPD). However, despite the power of RI‐XRPD methods, there remain immense challenges. In the present contribution, many of these challenges are highlighted, and their effect on the interpretation of RI‐XRPD data considered. A novel data processing technique is introduced for RI‐XRPD, through which the solvent‐free mechanochemical synthesis of an organic salt is followed as a case study. These are compared to ex situ studies, where notable differences are observed. The process is monitored over a range of milling frequencies, and a nonlinear correlation between milling parameters and reaction rate is observed. Kinetic analysis of RI‐XRPD allows, for the first time, observation of a mechanistic shift over the course of mechanical treatment, resulting from time evolving conditions within the mechanoreactor.

Chemistry has traditionally focused on photo‐, electro‐, and thermally induced transformations of molecules and materials. In recent years, however, attention has turned toward the application of mechanical energy to induce reactions: mechanochemistry. This field has developed widely,^1^, ^2^, ^3^ and numerous applications to the generation and processing of advanced organic, inorganic, and hybrid materials are known.^4^ For example, mechanochemical technologies have been successfully employed in the preparation of novel electronic and magnetic materials, the assembly of porous (e.g., metal‐organic and zeolitic‐imidazolate frameworks) and nanomaterials, and the generation of novel multicomponent crystals with improved physical properties.^5^, ^6^, ^7^ There is a growing need to understand the time‐dependence of these processes, and ascertain control over their temporal and spatial development. This is increasingly apparent for systems in which shear and impact forces exhibit different effects,^8^, ^9^ the rate of stressing affects the overall process,^10^, ^11^ the addition of small amounts of liquid alters the course of a reaction,^12^ and mixing^8^, ^13^ or stop‐start methodologies^8^ modify a reaction path. Recent studies have also demonstrated the importance of the stability of nanoparticles (product nuclei) in directing the mechanochemistry of molecular materials.^14^ Further impetus to understand and control these mechanochemical phenomena stems from their potential industrial applications as high‐yield, environmentally benign processes. This is of particular importance for industrial operations, where inadvertent or unexpected solid‐state transformations can have drastic impact on the performance of a material, with substantial legal, financial, and health safety ramifications. While theoretical studies have attempted to rationalize reactivity in ball mills,^14^, ^15^, ^16^ many of these processes remain largely out with the reach of modern ab initio techniques. Thus, the development of advanced experimental techniques is of critical importance.

A great advance for the study of mechanochemical dynamics has been the development of real‐time in situ (RI) monitoring by synchrotron X‐ray powder diffraction (XRPD),^17^, ^18^ complemented by Raman spectroscopy.^19^, ^20^ These developments offer significant advantages over conventional stop‐start methodologies. Particular benefit of RI methods surrounds the ability to identify short‐lived or unstable products, and allows monitoring of a continuously stressed system, without interim relaxation stages. Despite the numerous advantages of RI monitoring, one must be careful to acknowledge the many disadvantages and challenges it currently poses. A general issue of these techniques stems from the limited sampling area being monitored during mechanical treatment. Powder mixing is well known to be inhomogenous, particularly in mechanoreactors where various agitation‐induced demixing phenomena compete,^21^ and different sections of the reaction vessel may induce different products.^6^ Further, the quality of data attainable by RI methods is typically poor, and its interpretation difficult. This is a particular challenge for XRPD methods, where a large sample thickness, and multiple scattering points lead to artificial broadening of Bragg peaks. In this sense, the combined application of RI spectroscopic methods is a considerable advantage.^19^ Despite these issues, RI techniques have successfully identified intermediate species in mechanosynthesis, and attempts to derive mechanistic information have been made,^20^, ^22^ including under elevated global temperature.^23^ However, given the limitations of these techniques in time and spatial resolution, the sensitivity of these techniques, the lack of atomistic‐scale details of material properties under milling conditions, and the highly stochastic nature of milling processes, such mechanisms can only be speculative. That said, the ability of RI techniques to monitor the macroscopic evolution of a mechanochemical reaction is of undeniable importance, and the appropriate interpretation of these data crucial for extracting useful information.

In this paper general issues encountered with in situ monitoring of mechanochemical experiments, particularly with respect to data processing and experimental parameters are introduced. The interpretation of RI‐XRPD data is then discussed with emphasis on understanding the macroscopic evolution of milling processes. As a model system we have used γ‐glycine (γGly) + oxalic acid dihydrate (OAD). A mixture of these compounds is known to form two salt products on ball milling: bis(glycinium) oxalate (G_2_O) and glycinium semi‐oxalate (GO), **Scheme** **1**. This system is representative of many reactions that yield organic salts and cocrystals on cogrinding. It has been previously investigated using ex situ techniques,^24^ proving an interesting system with which to study potential competing product phases. Further, as this salt formation does not require external moisture or solvent for reaction, the complexity of the reactant mixture is greatly reduced.

**Scheme 1:**
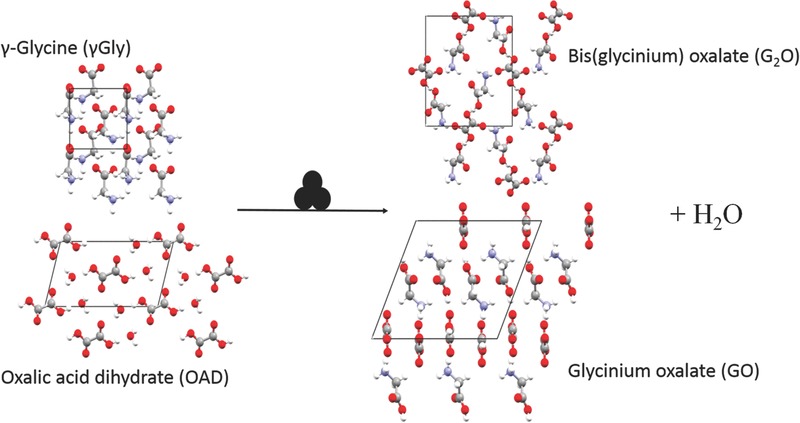
Mechanochemical reaction between γGly and OAD, to give G_2_O and GO salt products plus water.

One of the most challenging factors in RI‐XRPD is the associated artificial peak broadening and stochastic fluctuation of scattering intensities. In an ex situ experiment, the sample is immobile and its position with respect to the X‐ray beam is well defined and calibrated. By contrast, during an in situ experiment, the sample is in permanent motion within the milling jar, and thus the path of the immobile beam through the sample is time‐dependent. The stochastic motion of milling bodies within the milling jar further complicates in situ measurements. Hence, the sample that is analyzed by X‐ray diffraction varies not only because of a physical or chemical transformation, but also due to the stochastic motion of different particles flying in and out of the beam. While one can roughly estimate peak broadening to account for the thickness of the sample (with the maximum possible value determined by the jar diameter),^17^ its exact value is dynamic and dependent on the quantity and position of powder in the beam path at any moment in time. The ratio of phases present in this stochastically sampled material does not necessarily represent the composition of the sample as a whole. Such effects have been noted where the formation of different phases occurs at different jar sites.^9^ This proves very challenging for the processing of diffraction data. Of further complication is the considerable background associated with the milling jar, and the associated low signal‐to‐background intensity. Together, these issues render data processing a challenge, particularly by automated refinement strategies. Current methodologies for treating RI‐XRPD data^17^, ^23^ typically involve addition of a nonreactive calibrant into the sample, which is used to normalize and correct the data. In principle, this accounts for fluctuations in the quantity of diffracting sample, and either the ratio of sample to calibrant intensities, *I*_a_/*I*_cal,_ or automated Rietveld refinement (ARR) used to quantify the phase content and identify amorphous material. However, a number of issues surround this technique: (1) perfect mixing of the calibrant throughout the powder mixture must be assumed, and any deviation can be a source of erroneous amorphous content assignment, (2) the calibrant must be assumed not to act as a milling body, affect the rheology of the mixture, or to have any other chemical/physical effect on the milled powder, and (3) the use of *I*_a_/*I*_cal_ does not account for distribution of diffracted intensity across a broad signal. Even applying ARR in the absence of calibrant, while robust in many cases, encounters issues when faced with stochastic fluctuations in peak‐profile parameters, abnormal backgrounds, artificial peak splitting, or otherwise abnormally shaped peaks.^17^ Indeed, this led to issues in the processing of the present data, where phase fractions of the product phase were overestimated in the initial stages of the process and the profile dynamics were poorly represented in some cases. Instead, we suggest a hybrid technique (HT), combining careful Rietveld refinement of selected diffraction patterns and peak integration. We note that during milling, the powder is in continuous motion, and preferred orientation is negligible. This has been confirmed through Rietveld refinement. This method is summarized in **Figure** **1**Ia, and details are given in the Experimental Section. This type of methodology is expected to offer a general means by which difficult or fluctuating peak shapes may be treated, and can be extended to systems in which peak splitting is observed. Its main requirement is the existence of a high‐intensity, well‐resolved diffraction peak for each phase.

**Figure 1:**
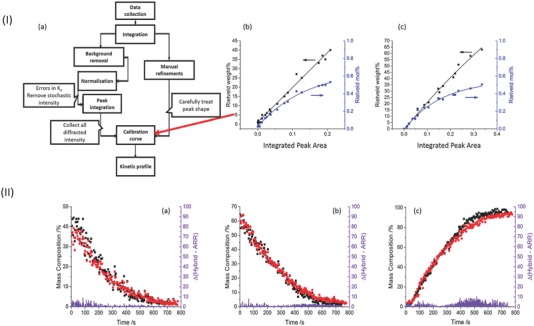
(Ia) Flow diagram for the hybrid technique (HT) to treat real time in situ diffraction data (see Supporting Information for details). Example calibration curves, Rietveld phase composition against integrated peak intensity, for γGly (Ib) and OAD (Ic) milled at 25 Hz. (II) Comparison of the hybrid methodology (black) with ARR (red). Absolute differences are shown in purple. Comparison is shown for 25 Hz milling for (IIa) glycine, (IIb) OAD, and (IIc) GO.

We were fortunate to obtain higher quality diffraction data than has previously been reported for RI‐XRPD measurements, and thus a careful comparison of the HT with ARR was possible. The phase composition profiles (PCP) for the mechanochemical reaction of γGly + OAD were processed by both techniques, Figure 1II. It is clear that in the present system—with high‐quality raw diffraction data—the HT yields near identical PCPs to those obtained through ARR methods, with the added benefit of modeling the early‐stage dynamics of GO formation (not possible by Rietveld methods). In addition, it was found that despite this higher‐quality data, ARR does not fully identify key features of the phase profiles of our system, particularly of OAD, and consideration of the full diffracted peak intensity is required for thorough analysis (see the Supporting Information). It is further worth noting that while ARR methods have great difficulty refining low mass fraction phases, the same is not true for the HT, which is only restricted by the accuracy of the calibration curve. One can extrapolate these calibration curves as necessary to composition regions inaccessible to ARR. The lower the quality of the raw data, the larger the benefits of applying the HT in lieu of ARR.

The phase composition of the powder—dα_*i*_/d*t*, where α_*i*_ is the transformation degree of phase *i*—was followed, milling at 25, 27.5, and 30 Hz, with the upper and lower limits restricted by the operating parameters of the mill. It is a common aim to vary mechanical energy input through variation of the milling frequency. However, in such cases, one must pay particular attention to the rheology of the system being treated, and the effects of frequency on the operation of the mill itself. In the present system, frequencies below 25 Hz caused the milling ball to roll. Thus, instead of inducing mechanical impact, the sample coated the milling ball. By contrast, at elevated frequencies, the powder was found to compact more rapidly at the milling jar ends; the quantity of free‐flowing powder that could be sampled by RI‐XRPD therefore greatly diminished. Such problems were minimized for the three frequencies chosen here.

Following the reaction profile by the HT, milling at all three frequencies showed the same general features; the two reactants are consumed, leading to formation of the stoichiometric product, GO, **Figure** **2**a–d. Only trace quantities of G_2_O are observed in the free‐flowing powder, and its lifetime shortens with increased milling frequency. We note that G_2_O does not appear to decompose on increased milling frequency as it can be made to become a major product by modifying the effective stoichiometry of the initial mixture (see the Supporting Information). Instead, this loss can be associated to either reaction with remaining OAD to form GO, or tableting of G_2_O against the vessel walls. The inability to distinguish between these possibilities further highlights the limited capabilities of current RI‐XRPD methods for mechanistic investigation of mechanochemical transformations. The limited formation of G_2_O contrasts with the same reaction in a drop‐hammer device where G_2_O appears in larger quantities.^24^ It is important therefore to remember that mechanochemical products can differ at the walls and ends (tableted powder) of a milling jar,^13^ and that current RI‐XRPD method monitors only the former. The G_2_O may therefore be formed not in the flowing powder or at the jar walls which are subject to shear,^13^ but exclusively at the jar ends on impact, as found in the drop‐hammer device and on ex situ sampling of ball milled samples (free and tableted powder) (see the Supporting Information). In this case, the G_2_O product remains undetected by the RI‐XRPD method. Such systems pose great risk to misinterpretation of RI data, and to industrial processes, where undetected seeds may lead to conversion of the sample on ageing.^25^

**Figure 2:**
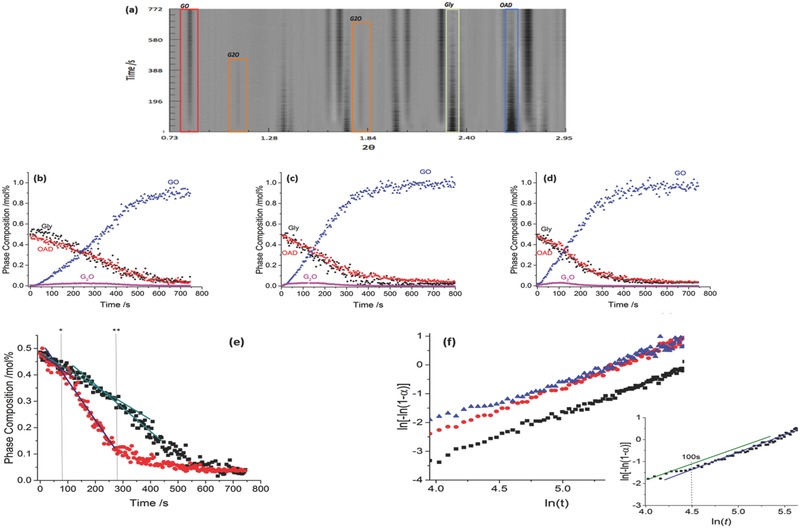
RI‐XRPD data for milling of γGly + OAD. a) 2D image of diffraction data for milling at 25 Hz. PCPs are shown for milling at b) 25 Hz, c) 27.5 Hz, and d) 30 Hz. e) OAD PCP at 25 Hz (black) and 30 Hz (red), with transitions marked by ** and *, respectively. f) Sharp–Hancock plots for the sigmoidal portion of GO production at 25 Hz (black), 27.5 Hz (red), and 30 Hz (blue). The stepped mechanism is highlighted for 30 Hz milling (inset).

In the present case, the inclusion of possible amorphous intermediate species has not been accounted for. First, we note that despite a plethora of literature surrounding the mechanochemistry of glycine, no evidence of an amorphous structure exists to date. Further it should be noted that milling is unable to amorphize hydrate materials unless performed at considerably depressed temperatures.^26^ This is due to the plasticizing effect of hydrate water molecules, and any potential amorphization is immediately lost to recrystallization. More generally, considerable efforts by Willart and Descamps^27^ into the nature of mechanically induced amorphization suggest that any detectable quantities of such phases can only occur if milling is performed below the glass transition temperature of these materials. The likelihood of long‐lasting, detectable amorphous phases is even further reduced if milling is conducted in humid or liquid assisted grinding (LAG) conditions.^26^, ^28^ Although the present study is not a typical LAG reaction, water is released as one of the products. Attempts to quantify organic amorphous intermediates by RI‐XRPD beyond qualitative assessment of powder patterns are likely erroneous, and loss of Bragg peaks is most probably due to decreased particle size, fusing scattering intensity to the high background, or loss of a phase from the beam path. Qualitative assessment of the XRPD profiles in the present work does not suggest any quantifiable amorphous content, and thus any associated error in the reaction profiles of Figure 2 will be within experimental error.

Prior to analysis of the PCPs, it is important to make note of the effect non‐normalized XRPD patterns can have on subsequent kinetic analysis. It is particularly striking to find that without normalization, the PCP for reactant species follows a typical first‐order exponential decay. This is not what is observed by ARR or the HT, having normalized the data. This suggests that, with time, the amount of free‐flowing powder reduces exponentially, as it becomes caked to the sides and walls of the milling vessel. Thus, in a similar situation, if one normalizes their XRPD profiles to the quantity of an internal standard, it must be assumed that all of this material remains evenly mixed and distributed between the free and compacted powder. Given the exponentially decaying PCP outlined in Figure S1.4.3.1 (Supporting Information), one might suggest that exponential removal of calibrant from the free‐flowing sample may erroneously introduce exponential kinetic models to the reaction PCPs.^29^ Such effects must be seriously considered, and are a tangential benefit of the HT, allowing one to directly probe the relative quantity of free‐flowing powder. Such effects are of great importance, as it is known that changes in ball‐to‐sample ratio can change the reaction profile.

With this in mind, it remains interesting to discuss the PCPs of the remaining free‐flowing powder, noting that the reaction in the tablet may differ. Inspection of the composition profiles for the molar consumption of γGly and OAD suggests that they do not reproduce well the conventional zeroth‐ or first‐order reaction profiles. Instead, an interesting feature is observed, most prominently at 30 Hz, Figure 2e.

Rather than exhibiting an exponential (first order) or completely linear (zeroth order) variation in PCP, the initial profile for OAD displays two linear segments, with a transition after ca.280 s at a milling frequency of 25 Hz and after ca. 100 s when at a milling frequency of 30 Hz. At these points, the observed rate constants increase from (6.29 ± 0.225) × 10^−4^ to (8.57 ± 0.358) × 10^−4^ αs^−1^ when milled at 25 Hz and from (7.49 ± 0.93) × 10^−4^ to (17.2 ± 1.30) × 10^−4^ αs^−1^(30 Hz), with both regimes suggesting zeroth‐order processes, known for other mechanochemical reactions.^30^ This transition corresponds to a roughly 30% increase in rate of OAD consumption at 25 Hz and over 100% increase by 30 Hz, thus it is more pronounced at higher milling frequencies. Only a very subtle transition is observed in the γGly PCP (see the Supporting Information), with less than 30% increase in γGly consumption rate observed at 30 Hz. This suggests that the nature of this transition directly affects the rate of OAD consumption, but only indirectly affects the rate of reaction of γGly. A possible mechanism involves the dehydration of OAD, and may result from a global increase in temperature or reduction of OAD particle size. Both are known to enhance the dehydration rate of OAD.^31^ It is known that solvate liquid is important in driving organic mechanochemical processes^23^, ^32^ and desolvation is believed to be responsible for formation of a fluidized (truly fluid or strongly disordered solid) intermediate state. In such a case, one would expect to see two stages of a reaction: one for the consumption of reactant material (fluidization), and a second corresponding to the nucleation/growth kinetics of the product. The rates of these two processes are not necessarily identical.

Following from this multistep model, where reactant consumption and product formation are treated independently, the formation of product can be formally described by a general equation, Equation (1)

(1) $$\alpha \;\;=\;\;1-e^{kt^{n}}$$

A specific case of this general equation, the Avrami–Erofeyev equation, has been applied previously^17^ to mechanochemical processes, and the various other forms of Equation (1) are known to fit any profile containing three basic stages: an induction period, an acceleration or growth phase, and a deceleration. When considering the transformation of a single phase into another, these stages are commonly interpreted as nucleation and nuclei growth.^33^ However, in the case of multicomponent reactions, with macroscopic analysis, a more appropriate interpretation follows as contact formation, reaction at contacts, and finally deceleration where the reaction rate becomes greater than new contact formation. The latter is due to reactant consumption. We note that unlike in solution, mechanochemical nucleation/growth is not continuous, and free nuclei will only continue to grow on subsequent impact with reactant material; stability of nanoscale nuclei is therefore important.^14^ The continuous growth of product suggests negligible impact of mixing on the current process.

Linearization under this formalism yields a Sharp–Hancock (SH) plot, Figure 2f, through which one can make general conclusions as to the evolution of the system composition. Constant *k* describes a critical point at which sufficient contacts exist to observe product, and constant *n* indicates the rate of product formation. We note that the conventional atomistic‐level interpretations of Equation (1) are not appropriate for analysis RI‐XRPD data, where sensitivity and time resolution are inadequate. Instead this macroscopic interpretation of Equation (1) offers quantification of the evolution and control of a mechanochemical process.

In the present system *n* = 1.618(22), 1.600(21), 1.469(25), and ln(*k*) = −9.762(125), −8.582(102), and −7.944(483) for 25, 27.5, and 30 Hz, respectively. The “macroscopic” constant (that is, one which does not correspond to an elementary chemical process), *n*, remains similar, with parallel SH plots, and ln(*k*) values varying largely across the series. This is confirmed through fitting of Equation (1) to the GO dynamics profiles (see the Supporting Information), where all constants are reproduced. This shift in ln(*k*) is interpreted as a decreased time to forming contacts on higher frequency milling. It is interesting to note the nonlinear relationship between milling frequency and vertical translation on the SH diagram.

With the most abrupt change in the OAD profile observed for the 30 Hz milling pattern, analysis of constant *k* for both OAD stages can be compared. The constant *n* increases slightly across this transition (1.336(51) and 1.580(43)), paralleling milling at 27.5 Hz. The shift in ln(*k*) [from −7.173(336) to −8.597(483), again identical to 27.5 Hz milling] suggest that this mechanistic shift may inadvertently impact on reactive contact formation. We note that this transition is only visible in the GO profile when it is very prominent in the OAD profile. Hence, there is only an indirect effect on GO formation. This is consistent with requirements for a multistage model of mechanochemistry. It is evident that the evolution of milling conditions is such that any mechanistic model cannot be built on the basis of constant conditions.

The collection and subsequent processing of mechanochemical data is an enormous challenge that has yet to be fully resolved. Despite the literature on the subject, there is to date no discussion of these challenges, or their impact on the interpretation of collected data. In this paper we have for the first time explicitly identified a number of these key issues in light of their effect on understanding mechanochemical data, and proposed alternative approaches where possible. Particular attention is paid to the difficulties associated with limited sampling during RI‐XRPD analysis, leading to considerable discrepancy in product distribution when compared to ex situ analysis. Through use of experimental PCPs obtained for a model system a novel data processing strategy has been discussed, and macroscopic mechanistic detail has been derived. This novel technique, based on a combination of Rietveld refinement and peak integration, has proved very promising for the processing of RI‐XRPD data. This has led to the first identification of time‐evolving kinetics in a mechanochemical co‐crystallization, although it remains unclear whether this evolution is due to changes in the free flowing powder, temperature, or particle size. It is evident that mechanochemical processes are considerably more complicated than often believed, and a complete understanding of the macroscopic dynamics is necessary to fully control these processes. However, to realize this, careful consideration must be given to the way in which these complex systems are studied. Only in this way can true insights into the fundamental mechanisms leading to the mechanochemical production of advanced materials be achieved.

## Experimental Section

*In Situ Milling*: Real time in situ milling experiments were conducted at the European Synchrotron Radiation Facility (ESRF), beam line ID11, experiment CH4313. Ball milling was done in a modified MM400 Retsch mill. For each reaction, 300 mg of stoichiometric mixture of oxalic acid dihydrate and glycine was used. Perspex milling jars (14.5 mL) were used^34^ with a single stainless‐steel ball (7 mm diameter). Monochromatic X‐ray of wavelength 0.141696 Å was used, and powder patterns were collected every 0.4 s. Data were averaged by summing 10 detector frames, giving a total time resolution of 4 s. Integration of 2D data was performed using the PyFAI azimuthal integration methodology.

*Hybrid‐Methodology*: To ensure sufficient sampling across the time domain, 20 integrated powder diffraction patterns were Rietveld refined using GSAS,^35^, ^36^ Figure S1.4.1.1 (Supporting Information), and quantitative phase information extracted, Figure S1.4.1.2 (Supporting Information). Patterns were selected to capture key phase evolutions throughout the process. It is noted that the refined quantities of G_2_O are within the error limits of the Rietveld refinement (<3 wt%), and its resulting dynamics profile is therefore an upper estimate of its phase composition throughout the process. Integrated data were subsequently background corrected using the Sonneveld–Visser algorithm^37^ in Powder3D.^38^ All patterns were numerically normalized to unity, in order to account for stochastic fluctuations in the quantity of diffracting sample, and the major peak of each phase integrated; both procedures performed using a custom‐designed program. Integration was performed using a trapezoidal algorithm on an evenly spaced grid. Integration is thus defined by the precision of experimental data points, collected here in 2θ steps of 0.00762. The refined compositions and integrated intensities were subsequently used to create calibration curves (see Figure S1.4.1.3, Supporting Information). It is noted that due to limitations in Rietveld refinement, a calibration curve for G_2_O was not possible. Instead, the raw dynamics profile was scaled to match the maximum Rietveld refined composition. This introduces an error to phase composition of no more than 3%. However, it is again worth noting a benefit over pure ARR techniques: processing data in this way continues to offer at least an approximate dynamics curve of the correct shape for low intensity phases. ARR, instead, produces only noise. For general use of this methodology, more complex systems are expected, in which multiple product phases appear in large quantities, that a cross‐correlation (e.g., a ratio between integrated phase peaks) term will be required in creating the calibration curves, or indeed in normalization. That is, to account for possible nonlinear scattering strengths between multiple products. In the current example, only one product is observed to any notable extent, and errors associated with neglecting this cross‐correlation term are expected to be less than inherent experimental error.

*Automated Rietveld Refinement*: Integrated data were used without background correction. ARR was performed in TOPAS.^39^ All lattice parameters were left to refine for γGly, OAD, and GO. However, due to abnormal peak shapes and background, profile parameters were fixed to manually refined values. It is noted that phase composition of G_2_O is within the error limits of ARR.

*Ex Situ Analysis, Drop Hammer Treatment*: Stoichiometric samples of 100 mg mixtures of γGly + OAD were subjected to impact treatment in a drop hammer device.^24^ A drop weight of 15.4 g was dropped from 17.5 cm. The frequency of successive impacts was 1.57 Hz. Samples were treated in a stainless‐steel anvil. New samples were produced for each experiment, and were treated for 0, 2.5, 5, 7.5, 10, and 12.5 min. Samples were removed from the anvil, all powder mixed, and analyzed by X‐ray powder diffraction.

*Ex Situ Analysis, Ball Milling*: Stoichiometric samples of 300 mg mixtures of γGly + OD were subjected to impact treatment in a Retsch Cryomill Ball Mill. A stainless‐steel ball (7 mm diameter), with stainless‐steel milling vessels (ca. 10 mL) were used. Milling was performed at 25 Hz to best reproduce in situ conditions. New samples were produced for each experiment, and were treated for 0, 1, 2, and 3 min. Samples were removed from jar, all powder mixed, and analyzed by X‐ray powder diffraction. All parameters, including powder quantity was chosen so as to most closely reproduce in situ experiments.

*X‐Ray Powder Diffraction*: All samples in ex situ experiments were analyzed by X‐ray powder diffraction. A STOE‐MP diffractometer (Cu *k*_α1_ = 1.54056 Å), equipped with a Ge (bent) monochromator and Mythen 1K detector. Scan step size of 0.135^o^, with total collection time of 16 min was used. Patterns were refined in GSAS.^35^, ^36^

## Conflict of Interest

The authors declare no conflict of interest.

## Supporting information

Supplementary
