# Supplementary material for: Challenges of Mechanochemistry: Is In Situ Real‐Time Quantitative Phase Analysis Always Reliable? A Case Study of Organic Salt Formation
Source: Adv Sci (Weinh). 2017 May 8;4(9):1700132. doi: 10.1002/advs.201700132 (PMC5604370; doi:10.1002/advs.201700132)
Supplement: Supplementary file 1 — Supplementary [file ADVS-4-na-s001.pdf]

Copyright WILEY-VCH Verlag GmbH & Co. KGaA, 69469 Weinheim, Germany, 2017.

ADVANCED SCIENCE
Open Access

Supporting Information

for *Adv. Sci.,* DOI: 10.1002/advs.201700132

Challenges of Mechanochemistry: Is In Situ Real-Time Quantitative Phase Analysis Always Reliable? A Case Study of Organic Salt Formation

Adam A. L. Michalchuk, Ivan A. Tumanov, Sumit Konar, Simon A. J. Kimber, Colin R. Pulham,* and Elena V. Boldyreva**

Supplementary Material:
Challenges of Mechanochemistry: Is *in situ* Ream-Time Quantitative Phase Analysis Always Reliable?.**

Adam A.L. Michalchuk,[a,b,c] Ivan A. Tumanov,[b,d] Sumit Konar, [a] Simon A.J. Kimber,[e] Colin R. Pulham*[b,c] and Elena V. Boldyreva*[d]

Contents:

S1. Materials and Methods………………………………………………………….. 2

S1.1 Materials

S1.2 *In situ* Mechanochemistry

S1.3 *Ex situ* Mechanochemistry

S1.4 Data Processing

S2. ARR vs. Hybrid Technique………………………………………………………. 5

S3. *Ex Situ* Mechanochemistry……………………………………………………… 6

S4. Solid State Dynamic Profiles…………………………………………………… 7

S5. Non-Linearity in Mechanochemistry……………………………………… 9

S6. Practicalities of Mechanochemical Experiments…………………….. 10

REFERENCES………………………………………………………………….... 12

WILEY-VCH

S1. Materials & Methods:

S.1.1 Materials: Oxalic acid dihydrate (OAD; Reakhim, Russia) and γ-glycine (γGly; recrystallized from ICN Biomedicals by ammonia vapour[1]) were used without further purification. Both products, glycinium oxalate (GO) and bis(glycinium)-oxalate (G_2O) were prepared *in situ* in all experiments. Where required for *ex situ* studies, α-glycine (αGly; ICN Biomedicals) was used without further purification.

S1.2 *In Situ* Mechanochemistry: Real time *in situ* milling experiments were conducted at the European Synchrotron Radiation Facility (ESRF), beam line ID11, experiment CH4313. Ball milling was done in a modified MM400 Retsch mill. For each reaction, 300 mg of stoichiometric mixture of oxalic acid dihydrate and glycine was used. Perspex milling jars (14.5 mL) were used[2] with a single stainless-steel ball (7 mm diameter). Monochromatic X-ray of wavelength 0.141696 Å was used, and powder patterns were collected every 0.4 s. Data were averaged by summing 10 detector frames, giving a total time resolution of 4 s. Integration of 2D data was performed using the PyFAI azimuthal integration methodology.

S1.3 *Ex Situ* Mechanochemistry: *Ex situ* studies were performed in both a custom-built drop-hammer device[3] and Retsch Cryo-Mill.

S1.3.1 Drop Hammer: Stoichiometric samples of 100 mg mixtures of γGly + OAD were subjected to impact treatment in a drop hammer device.[3] A drop weight of 15.4 g was dropped from 17.5 cm. The frequency of successive impacts was 1.57 Hz. Samples were treated in a stainless-steel anvil. New samples were produced for each experiment, and were treated for 0, 2.5, 5, 7.5, 10 and 12.5 minutes. Samples were removed from the anvil, all powder mixed, and analysed by X-ray powder diffraction.

S1.3.2 Ball Milling: Stoichiometric samples of 300 mg mixtures of γGly + OD were subjected to impact treatment in a Retsch Cryomill Ball Mill.[3] A stainless-steel ball (7 mm diameter), with stainless-steel milling vessels (*ca* 10 mL) were used. Milling was performed at 25 Hz to best reproduce *in situ* conditions. New samples were produced for each experiment, and were treated for 0, 1, 2, and 3 minutes. Samples were removed from jar, all powder mixed, and analysed by X-ray powder diffraction. All parameters, including powder quantity was chosen so as to most closely reproduce *in situ* experiments.

S1.3.3 X-ray Powder Diffraction: All samples in *ex situ* experiments were analysed by X-ray powder diffraction. A STOE-MP diffractometer (Cu $k_{\alpha 1}$ =1.54056 Å), equipped with a Ge (bent) monochromator and Mythen 1K detector. Scan step size of 0.135^{o}, with total collection time of 16 min was used. Patterns were refined in GSAS.[4,5]

WILEY-VCH

S1.4 Data Processing:

S1.4.1 Hybrid-Methodology: To ensure sufficient sampling across the time domain, 20 integrated powder diffraction patterns were Rietveld refined using GSAS,[4,5] Figure S1.4.1.1, and quantitative phase information extracted, Figure S1.4.1.2. Patterns were selected so as to capture key phase evolutions throughout the process. We note that the refined quantities of G_2O are within the error limits of the Rietveld refinement (< 3 weight%), and its resulting dynamics profile therefore an upper estimate of its phase composition throughout the process. Integrated data were subsequently background corrected using the Sonneveld-Visser algorithm[6] in Powder3D.[7] All patterns were numerically normalisation to unity, in order to account for stochastic fluctuations in the quantity of diffracting sample, and the major peak of each phase integrated; both procedures performed using a custom-designed programme. Integration was performed using a trapezoidal algorithm on an evenly spaced grid. Integration is thus defined by the precision of experimental data points, collected here in steps of 0.00762 2θ. The refined compositions and integrated intensities were subsequently used to create calibration curves, Figure S1.4.1.3. We note that due to limitations in Rietveld refinement, a calibration curve for G_2O was not possible. Instead, the raw dynamics profile was scaled to match the maximum Rietveld refined composition. This introduces an error to phase composition of no more than 3%. However, it is again worth noting a benefit over pure ARR techniques: processing data in this way continues to offer at least an approximate dynamics curve of the correct shape for low intensity phases. ARR, instead, produces only noise. For general use of this methodology, note: we expect for more complex systems, in which multiple product phases appear in large quantities, that a cross-correlation (e.g. a ratio between integrated phase peaks) term will be required in creating the calibration curves, or indeed in normalisation. That is, to account for possible non-linear scattering strengths between multiple products. In the current example, only one product is observed to any notable extent, and errors associated with neglecting this cross-correlation term are expected to be less than inherent experimental error.

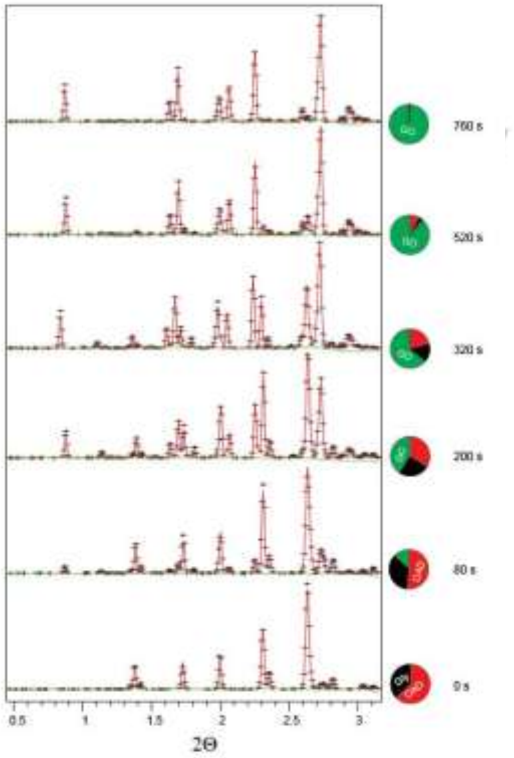

Fig S1.4.1.1: Sample Rietveld refinements for *in situ* real-time diffraction data.

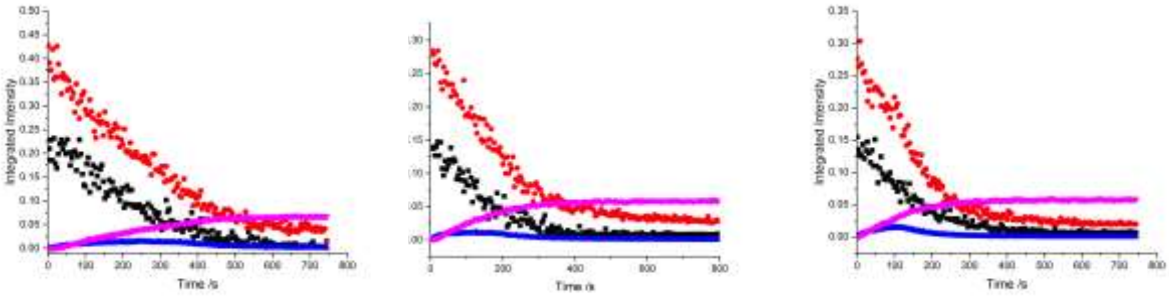

Fig. S1.4.1.2.: Integrated peak intensities for 25 Hz (left), 27.5 Hz (middle) and 30 Hz (right). In each case, all four phases are shown: γGly (black), OAD (red), G_2O (blue) and GO (pink).

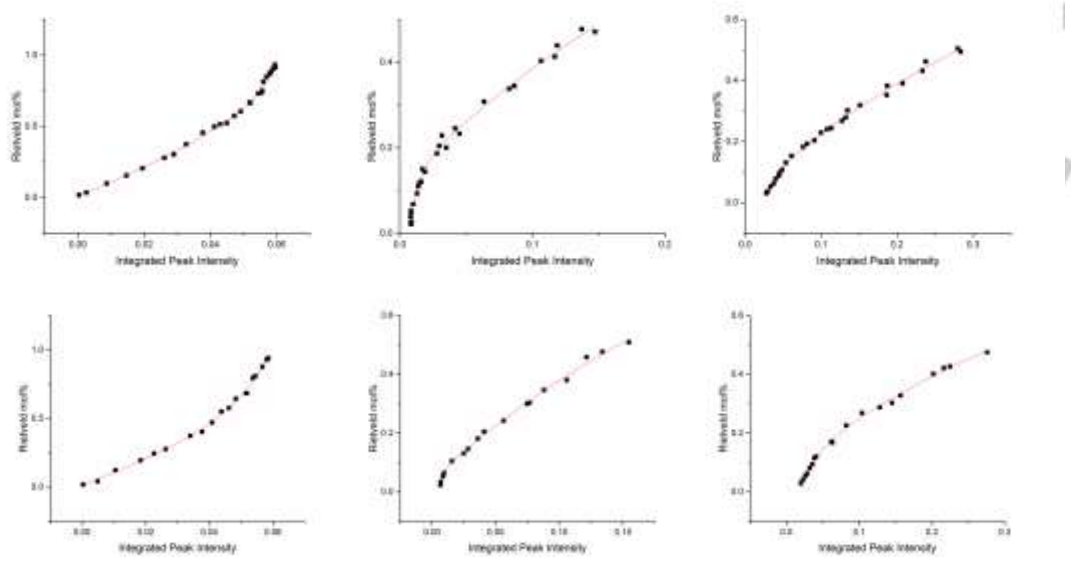

Fig. 1.4.1.3: Calibration curves for 27.5 Hz (top) and 30 Hz (bottom). In all cases, that for GO (left), γGly (middle) and OAD (right) are given.

S.1.4.2 Automated Rietveld Refinement (ARR). Integrated data were used without background correction. ARR was performed in TOPAS[8]. All lattice parameters were left to refine for γGly, OAD and GO. However, due to abnormal peak shapes and background, profile parameters were fixed to manually refined values. We note that phase composition of G_2O is within the error limits of ARR.

S1.4.3 General Notes on Data Processing for in Situ Diffraction. In view of studying mechanochemical mechanisms, it is of interest to highlight the normalization stage of Section 1.4.1. It is often assumed that the quantity of powder being treated throughout a milling process remains constant; that is that the ball:powder ratio remains constant. Changes to the quantity of powder present, or similarly the packing of powder,[9] drastically change the nature of mechanical treatment. Comparing the non-normalized to normalized dynamics profiles for the two reactant species, γGly and OAD, two considerably different profiles are seen, Figure S1.4.3.1. In the first, reactant is consumed exponentially, while in the latter, this becomes linear. This exponential loss observed in the non-normalized patterns does not reflect physical conversion of the material, but instead loss of free-flowing powder. Thus any sampling through *in situ* real time XRPD samples an exponentially smaller portion of the powder mixture, and may therefore give erroneous insight into the *true* nature of the mechanochemical conversion.[9] Of further importance are the implications to mechanical treatment itself. First, compaction leads to changes in the ball:powder ratio at impact zones and second, a compact powder is subject to considerably higher forces on impact, where powder compression is no longer an alternative energy dissipation channel. Powder compaction is a highly ubiquitous, seldom reported and often unavoidable occurrence in organic mechanochemistry, where liquids (atmospheric, crystalline or added) play a central role. Such considerations are therefore critical when investigating mechanochemical mechanisms by ball milling.

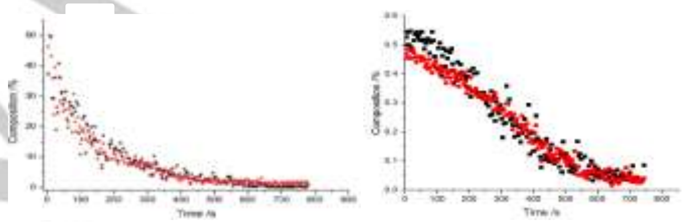

Fig S1.4.3.1: Comparing the γGly (red) and OAD (black) profiles for non-normalised (left) and normalised (right) data.

S2 ARR *vs* Hybrid Technique:

S.2.1: OAD dynamics.

While qualitatively the ARR and hybrid techniques produce the same profiles, see Fig 2 (in text), a comparison of the OAD dynamics profile for the two techniques is surprising. While the hybrid technique reveals an obvious transition in the OAD dynamics profile, it is considerably subtler in the ARR-deduced curve, most clearly seen for milling at 30 Hz, Figure S2.1.1. It is believed to be the result of restrictions imposed on the ARR, such as restricted profile parameters, which were required to ensure successful ARR.

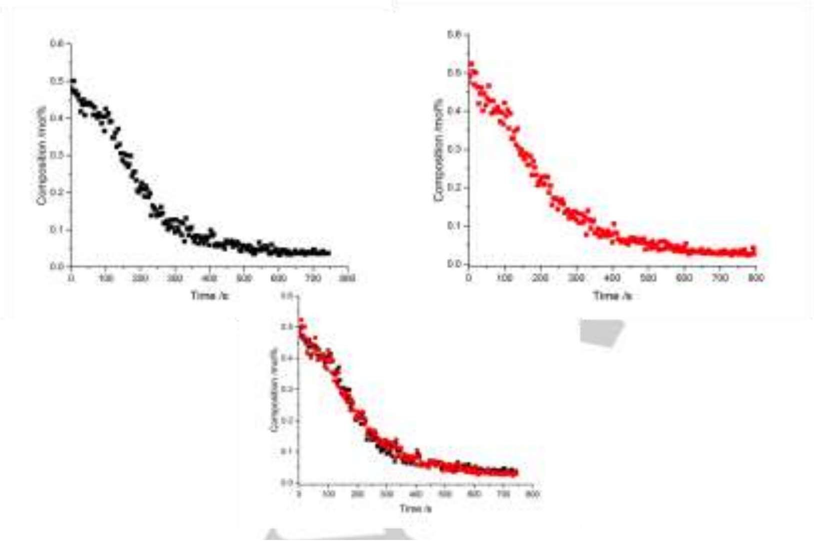

Fig.S2.1.1: Comparing OAD profiles for milling at 30 Hz. Profile generated from the hybrid method (black) and ARR (red) are shown. It is clear that both techniques offer the same general trend, however that by the hybrid technique is more revealing of a switch in dynamic profile.

To ensure this was not an artefact of the data-processing methodology, a number of possibilities can be considered. First is the data integration stage, where one might expect that the sudden introduction of a new phase may skew the normalized intensities. However, it is clear that, in all cases, the point at which the OAD mechanistic transition is observed does not correspond to the introduction of any new phase, Figure S1.4.1.2 and Fig 3 (in text).

An alternative explanation may rest in the migration of the integrated peak, outwith the integration zone. As is clear from Figure S.2.2.2, however, this is not the case. Integrated peak remains within the integration zone at all times, and no other peaks are seen to encroach.

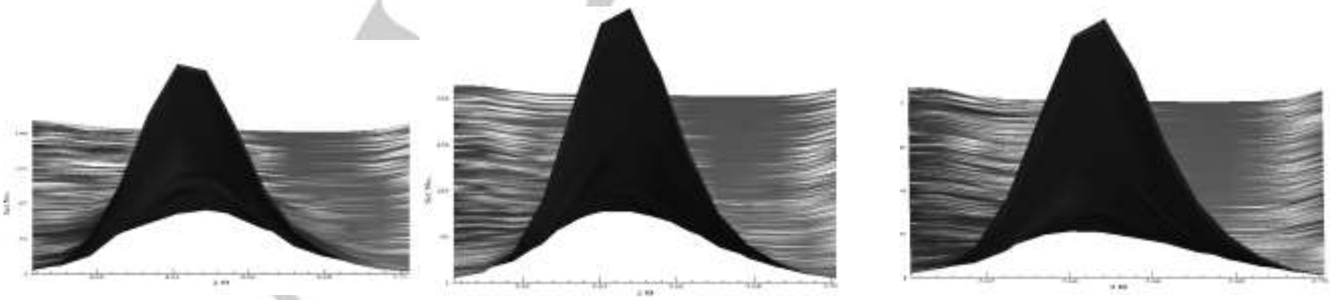

Fig S2.2.2: Non-normalized peak integration zone for OAD at 25 Hz (left), 27.5 Hz (middle) and 30 Hz (right).

S3. *Ex Situ* Mechanochemistry

To mimic the effects observed in tableted powder found at the ends of a milling jar, powder samples were treated in a drop-hammer device. This mimics the lack of mixing experienced by strongly tableted powders in mechanochemistry. The general progression of the reaction is the same as observed *in situ* in the ball mill: reactant species decrease with time, and the product phases increase, Figure S3.1. In contrast, however, the quantity of G_2O is substantially higher than the *ca.* 3 mol% observed with *in situ* sampling of the ''free'' powder.

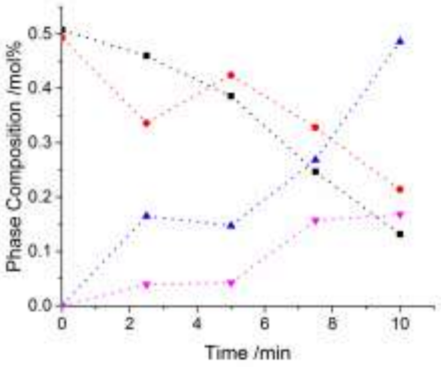

Fig S3.1: Phase composition of a stoichiometric mixture of γGly + OAD induced by drop-hammer, sampled *ex situ*. Curves are shown for OAD (red), γGly (black), GO (blue) and G_2O (pink).Dotted lines are shown as guides.

The discontinuous evolution of each phase is due to the creation of new samples for each experiment. Thus, mixing will differ slightly between samples. The overall trend, however, is significant and demonstrates the very different evolution of the powder in a tableted sample.

Ex situ sampling of a ball milling reaction reveals much the same trend: that, if the tableted powder is sampled, the G_2O quantity extends well above the 3 mol% observed by *in situ* monitoring, Figure S3.2. In the present example, powder mixtures were only run for up to 3 minutes' milling, yet the G_2O composition (including tableted powder) exceeds 10 mol%.

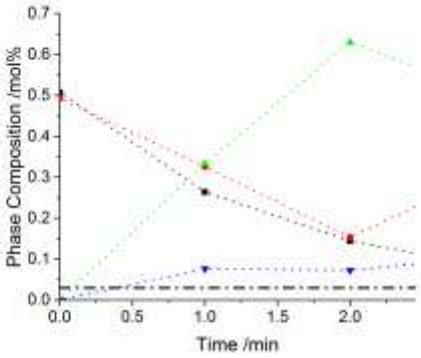

Fig S3.2: Phase composition of a stoichiometric mixture of γGly + OAD induced by milling, sampled *ex situ* and including tableted material. Curves are shown for OAD (red), γGly (black), GO (blue) and G_2O (pink). Dotted lines are shown as guides. The black dotted line is the upper limit for G_2O formation observed with i*in situ* monitoring.

S4. Solid State Dynamic Profiles

We first note that with current limitations of real time *in situ* XRPD technologies, one cannot justify use of the word *kinetics* in its traditional sense. That is to say that kinetics has traditionally been used to describe fundamental, elementary processes on the atomic/molecular scale. While the mathematical formalisms derived for these fundamental kinetic processes are universal, their interpretation must be re-considered in light of limitations in resolution. In that regard, it is more appropriate to discuss mechanochemical *dynamics*, where interpretation of formal equations reflects the macroscopic mechanisms associated with particle-particle interactions. The same must be said for the application of traditional solid-state kinetics models, such as the Avrami-Erofeyev equations, and related diffusion-based models. While the mathematical formalisms of these models can be used universally, where they accurately reflect the shape of the time-evolving data, interpretation of their derived constants in light of the original atomistic models would be erroneous. Many introductory texts are available on solid state kinetics, including Refs.10 and 11.

In order that bulky organic molecules are able to interact beyond the 1:1 interaction at particle surfaces (which is required to reach a critical nucleus size), the mobility of these materials must be greatly enhances. This requires some form of fluidisation, whether amorphisation, dissolution, melting, vapourisation or otherwise. To this end, one must consider *at least* two different mechanisms when treating the dynamics of mechanochemistry: (1) the consumption of reactant phases, which is to occur through melting, amorphisation, vapourisation or dissolution, and (2) the nucleation and growth of the reactant phase. The use of fluid-phase kinetics for the consumption of reactant has been used previously[12] for mechanochemical processes. To many extents this is valid, as both processes are largely dependent on multi-body collisions. Its use in the present work is done in as much as it highlights the dynamical features of the curves well, and interpretation is made in light of particle contacts/collisions.

It is more reasonable to employ nucleation/growth kinetics for the product phase. The GO curve is therefore interpreted in light of the general equation, Equation 1. Sharp-Hancock plots are simply a linearization of general Equation 1,[13]

$$\alpha = 1 - e^{-kt^n} \qquad \textbf{Equation 1.}$$

$$\ln(1-\alpha) = -kt^n$$

$$\ln(-\ln(1-\alpha)) = \ln(k) + n\ln(t)$$

This linearization is general, and its use not reliant on any specific kinetic model. In the present case, where macroscopic dynamics of mechanochemistry are concerned, it is most logical to interpret *k* as a critical mixing constant, which describes the time required for sufficient contacts to be formed in order to observe product formation (note that this constant will be resolution dependent, and is thus only meaningful when comparing identical experimental set-ups). Constant *k* is therefore indicative of mixing rate, and can be used to compare mixing parameters, including the effects of pre-mixing. The formal constant, *n*, is then indicative of the rate of product growth at contacts. Fits of the GO dynamics curve, Figure S4.1, yield values for k and n that correlate well to those reported in the main text, derived from Sharp-Hancock plots. We note that critical time t_c where $d^2\alpha/dt^2 = 0$ is a characteristic point, where the rate of product formation becomes greater than the formation of new contacts, and the deceleration regime begins, Figure S4.2. This offers an intriguing method to analyse mechanochemical dynamics, where the inflexion point may offer a means to study mixing rates and particle size effects, where more contacts become available more quickly. Here, this inflexion point is seen much more

rapidly for 30 Hz than 27.5 Hz. It is further worth noting that the second feature observed in Figure S4.2 directly correlates to the time at which the mechanistic shift is seen in the dynamics profiles. Additional features require future investigation.

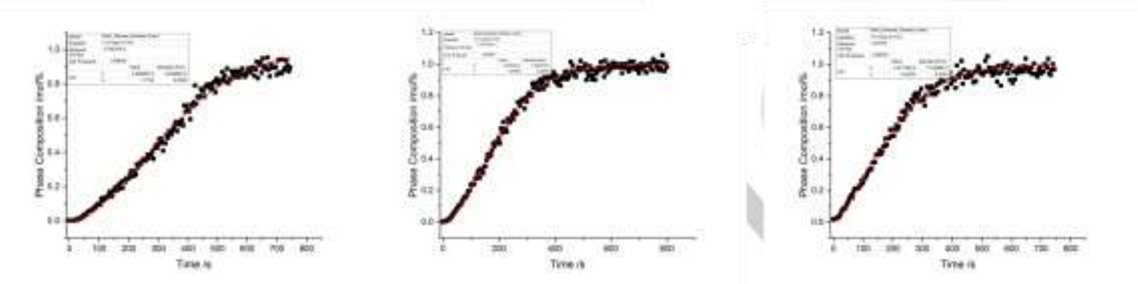

Fig S4.1: Fits of GO dynamics curves to general equation, Equation 1. Fits are given for 25 Hz (left), 27.5 Hz (middle) and 30 Hz (right). In each case, the resulting constants correlate well with those derived from Sharp-Hancock fits in text.

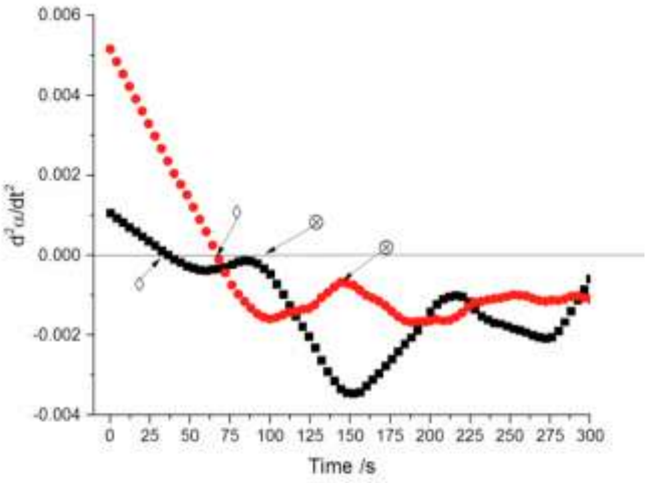

Fig. S4.2: Second derivative of the GO dynamics profiles. The inflexion point of the sigmoidal curve is seen (◆) along with the mechanistic transition point (⊗). Curves are shown for 27.5 Hz (red) and 30 Hz (black).

The mechanistic shift observed in the zeroth order OAD profile is also observed in the 1st order Gly profile (ln(Gly)), Figure S4.3. This suggests that any consequence of this transition plays an indirect role on consumption of Gly. The observed transition at approximately 100 s in Gly is consistent with that observed in OAD.

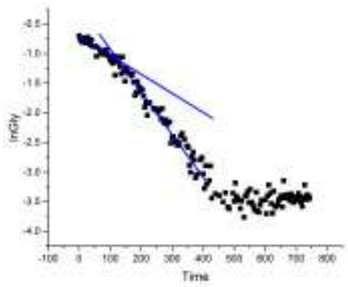

Fig. S4.3: First order profile of Gly milled at 30 Hz. Lines are drawn to guide the eye.

S5. Non-Linearity in Mechanochemistry

Increasing the frequency from 25 Hz to 27.5 Hz and further to 30 Hz is met with a non-linear shift in the rate of the milling reaction. For example, the approximate time at which the accumulation of GO plateaus (Acc-Plat Transition), increases roughly 25% from 25 Hz to 27.5 Hz, yet by nearly 65% on increasing from 25 Hz to 30 Hz, Table T5.1. It is not obvious why such non-linearity exists, but is likely due to heating, particle size effects or the accumulation of mechanical energy.

TABLE T5.1: Relations between milling frequency and time to the accumulate-plateau transition (Acc-Plat; A-P).

Frequency /Hz	Increase w.r.t 25 Hz /%	Acc.-Plat. Transition /s	Increase (A-P) w.r.t 25 Hz /%
25	0	450	0
27.5	10	325	23
30	20	280	63

WILEY-VCH

S6. Practicalities of Mechanochemical Experiments

Very many parameters can affect the result of a mechanochemical process, including moisture, temperature, milling ball size, particle size and shape, powder mixing, and many others. In many cases, such factors are very difficult, or impossible, to control, particularly when concerning particle characteristics, which change with time on milling. While it is of the utmost importance to understand how each of these parameters in turn affects a mechanochemical process, much remains to be understood about the mechanochemical process itself, under apparently constant conditions. The present work has highlighted how even under consistent controllable conditions, a mechanochemical process may not proceed as straightforwardly as believed. To highlight the immense additional complexity of planning mechanochemical procedures, we conducted the reaction under various milling frequencies, as highlighted in the preceding sections.

S6.1 Practical Limitations on Milling Frequencies

Typically, laboratory-scale ball mills are limited to a maximum frequency of 25-30 Hz, as is the case with the MM400 Retsch ball mill. Thus, no milling could be performed above 30 Hz in the present work. It is very easy to reduce milling frequency, as is reportedly done. Less often, however, is one able to visually observe the state of the powder mixture under various milling conditions. Below *ca* 25 Hz, it was found that the milling body is no longer propelled through the milling vessel, but instead remains within the central shaft of the milling vessel, ‘’rolling’’ back and forth as the vessel moves around it. This results in a ‘’snow balling’’ effect, Figure S6.1. In such cases, the reaction cannot be expected to proceed in a typical mechanochemical fashion, if at all, and thus milling frequencies below 25 Hz could not be reliably tested.

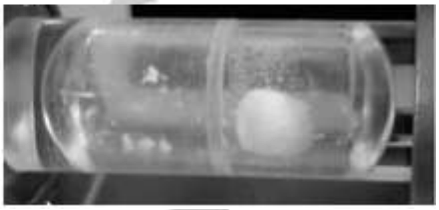

Fig S6.1: Powder ‘’snow-balling’’ within the milling jar at 20 Hz.

While within this work the milling frequency is varied by only 5 Hz, this amounts to an extra 300 impacts per minute, or an extra 300 possible reaction events *every minute*. It is therefore not surprising to find that such small adjustments to milling frequency yield different reaction rates. This is analogous to increasing the temperature of a gas or solution phase reaction, where reaction rate $\propto \sqrt{RT}$, that is, the collision frequency.

S6.2 Modifying Initial Particle Size

Particle size is often ignored as a control parameter, as the milling process is believed to immediately micronize the initial sample. In the main body of the present study, as with most reported mechanochemical investigations, particle size was taken as available from the supplier, aiming to highlight complexities with current methods of conducting mechanochemical experiments.

In related work, we performed initial assessment of particle size effects on milling processes. Here, micronized glycine was mixed with OAD and milled at 25 Hz. The resulting product profiles demonstrate considerably different compositions, Figure S6.2a. Where micronized glycine is used as initial reagent, considerably higher quantities of the 2:1 G_2O product are observed. We therefore propose that a mechanochemical process is *not* driven by the overall composition of the mixture, as is the case with solution phase processes, but is instead determined by the local reaction composition at the impact zone, Figure S6.2b. Further investigation into controlling reactions through particle size is therefore of great importance to the continued development of mechanochemistry.

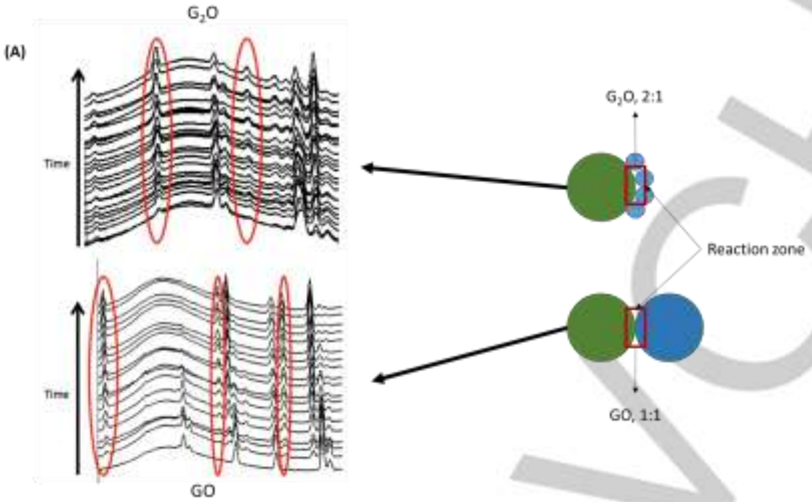

Fig S6.2: Comparing reaction profiles using (top) micronized glycine and (bottom) commercial glycine particle size. In the former, considerably higher quantities of the 2:1 product are observed, while the 1:1 salt is the main product of the latter. (A) The time resolved XRPD are shown, with major product phases indicated. (B) A schematic representation of the proposed reaction zone for each case.

WILEY-VCH

References

1. E.V. Boldyreva, V. A. Drebushchak, T.N. Drebushchak, I.E. Paukov, Y.A. Kovalevskaya and E.S. Shutova, *J. Therm. Anal. Cal.,* 2003, 73, 419-428.

2. I. Halasz, S. A. J. Kimber, P. J. Beldon, A. M. Belenguer, F. Adams, V. Honkimäki, R. C. Nightingale, R. E. Dinnebier, T. Friščić, Nature Protocols, 2013, 8 (9), 1718-1729

3. I. A. Tumanov, A. F. Achkasov, E. V. Boldyreva, and V. V. Boldyrev, *CrystEngComm*, 2011, **13**, 2213.

4. A. C. Larson and R. B. Von Dreele, 2000, 86–748.

5. B. H. Toby, *J. Appl. Cryst.*, 2001, **34**, 210–213.

6. E. J. Sonneveld and J. W. Visser, *J. App. Chem.*, 1975, **8**, 1–7.

7. B. Hinrichsen, R. E. Dinnebier, and M. Jansen, *Z. Kris.*, 2004, **23**.

8. Coelho, A. A. (2015). TOPAS Academic. Version 4. http://www. topas-academic.net.

9. A. A. L. Michalchuk, I. A. Tumanov, and E. V Boldyreva, *CrystEngComm*, 2013, **15**, 6403–6412.

10. VV Boldyrev, *Methods for Studying Kinetics of Thermal Decomposition of Solids (russ.)*, Tomsk, Russian Federation, 1958

11. B. Delmon, *Introduction to Heterogenous Kinetics*, Technip, Paris, 1969

12. X. Ma, W. Yuan, S. E. J. Bell, and S. L. James, *Chem. Comm.*, 2014, **50**, 1585.

13. J. D. Hancock and J. H. Sharp, *J. Am. Ceramic Soc.*, 1972, **55**, 74–77.

WILEY-VCH
